# Physical activity and the risk for gestational diabetes mellitus amongst pregnant women living in Soweto: a study protocol

**DOI:** 10.1186/s12905-016-0345-z

**Published:** 2016-10-18

**Authors:** Tasneem Khan, Shelley Macaulay, Shane A. Norris, Lisa K. Micklesfield, Estelle D. Watson

**Affiliations:** 1 Centre for Exercise Science and Sports Medicine, School of Therapeutic Sciences, Faculty of Health Sciences, University of the Witwatersrand, Johannesburg, South Africa; 2 MRC/Wits Developmental Pathways for Health Research Unit, Department of Paediatrics,School of Clinical Medicine, Faculty of Health Sciences, University of the Witwatersrand, Johannesburg, South Africa; 3 Division of Human Genetics, School of Pathology Faculty of Health Sciences, University of the Witwatersrand and the National Health Laboratory Service, Johannesburg, South Africa

**Keywords:** Physical activity, Gestational diabetes mellitus, Pregnancy

## Abstract

**Background:**

Over the past decade the prevalence of gestational diabetes mellitus (GDM) has increased rapidly in both developed and developing countries and has become a growing health concern worldwide. A recent systematic review highlighted the paucity of data available on the prevalence and potential burden of GDM in Africa, which was emphasised by the fact that only 11 % of African countries were represented in the review. In South Africa, the prevalence of GDM remains unknown, although one would estimate it to be high due to urbanisation and the growing obesity epidemic. In addition, the association between physical activity (PA), sedentary behaviour (SB) and GDM is not well understood in this population. The aim of the proposed research is to determine whether there is an association between physical activity, sedentary behaviour and risk for GDM in pregnant black women living in urban Soweto in South Africa.

**Methods/Design:**

This prospective cohort study of 80 participants will include pregnant women from Soweto enrolled into the Soweto First 1000 Days Study (S1000) at the MRC/Wits Departmental Pathways for Health Research Unit (DPHRU) based at the Chris Hani Baragwanath Academic Hospital in Soweto, South Africa. Women will be enrolled into the S1000 Study at <14 weeks gestation, and baseline demographic and anthropometric measures will be taken at 14–18 weeks gestation (visit 1). In addition, participants will complete the Global Physical Activity Questionnaire (GPAQ) to measure self-reported physical activity and will be given an ActiGraph accelerometer to wear for seven days to measure habitual physical activity at 14–18 weeks gestation (visit 1), and at 28–33 weeks gestation (visit 3). At visit 2 (24–28 weeks gestation) an oral glucose tolerance test (OGTT) will be conducted.

**Discussion:**

Physical activity during pregnancy has been associated with minimum risk to a pregnancy and may play a role in improving glucose metabolism and therefore decreasing risk for GDM. This is particularly pertinent to assess amongst black South African women who are a potentially high risk population due to the high prevalence of obesity and type 2 diabetes (T2D). The findings of the study will assist in developing targeted interventions as well as feasible healthcare strategies.

## Background

### Introduction

Gestational diabetes mellitus (GDM) is a significant and growing health concern worldwide [1]. It is defined as glucose intolerance of variable severity with onset, or first recognition, during pregnancy [2]. Certain populations are more susceptible to developing this condition due to genetic, social and environmental factors [1, 2]. Even though GDM is considered a temporary condition that usually recedes after child birth, if uncontrolled it can have substantial negative long-term effects for both the baby and the mother [1]. Recent data suggests that early detection and treatment of this condition can reduce the adverse maternal and offspring outcomes [3].

In light of the recently published Hyperglycaemia and Adverse Pregnancy Outcomes (HAPO) study [3], the International Association of Diabetes and Pregnancy Study Group (IADPSG) formulated new guidelines for screening and diagnosing diabetes in pregnancy [4]. Despite this, universal screening of pregnant women for GDM is not standard practice around the world, especially in developing countries [5]. In South Africa the national prevalence of GDM remains unknown, with only four studies reporting on regional prevalence figures, ranging from 1.8 % in urban to 8.8 % in rural areas [6–9]. As the prevalence of obesity and T2D are increasing in South African urban areas [10] it is plausible that the prevalence of GDM may increase [11, 12].

Physical activity has been shown to reduce glucose levels, improve insulin sensitivity and reduce the risk of developing of T2D independent of obesity [13, 14]. The association between physical activity (PA), sedentary behaviour (SB) and GDM risk is not well understood and is yet to be researched within an African context. Tobias et al. [15] concluded from their systematic review that physical activity prior to, or in early pregnancy, is associated with a significantly lower risk of GDM.

The aim of this study is to (1) objectively and subjectively measure physical activity in pregnant women in the second and third trimester of pregnancy; (2) Assess the association between physical activity levels in the second trimester and glucose tolerance; (3) Assess the association between physical activity levels in the second trimester and insulin sensitivity.

### What does the evidence say?

During pregnancy, glucose metabolism differs from the non-gravid state in order for the mother to meet her own needs as well as the energy needs of the growing fetus. Insulin is the key regulator of maternal metabolism and insulin sensitivity changes during gestation [16]. Women who are unable to respond suitably to the metabolic changes that occur during pregnancy are at a higher risk of hyperglycaemia and developing GDM [4].

Recent evidence amongst many ethnic groups has shown an approximate 50 % increase in GDM prevalence over the last 20 years [1]. It is estimated that the prevalence of GDM in Africa is approximately 5 % which is 2.5–17 % greater than some high income countries [17]. Women diagnosed with GDM are at higher risk of developing T2D in later years, with approximately 17–63 % of women developing it within 5 years after delivery [18]. Women with GDM are also thought to be at higher risk of developing pre-eclampsia and hypertension during their pregnancies [19]. Vohr and Boney [20] have shown that women who have a history of GDM and obesity have a significantly greater risk of developing metabolic syndrome than women with no history of GDM or obesity.

Furthermore, Yogev and Visser [21] have shown that children born to mothers with GDM have an increased risk of early obesity, T2D in adolescence, and development of metabolic syndrome in early childhood [21]. It has also been found that fetuses exposed to a high glucose environment are more likely to be macrosomic (>4 kg), and are more likely to experience other medical complications after delivery, such as infant respiratory distress syndrome, cardiomyopathy, hypocalcaemia, hypoglycaemia and polycythaemia [21–23].

At present there is no consensus on the optimal strategy for screening of GDM risk and identifying women with GDM. The most accepted diagnostic test for GDM is the Oral Glucose Tolerance Test (OGTT) performed at 24–28 weeks gestation [3]. The availability of screening women for GDM differs from country to country, and even within countries. In Italy, Turkey and Sweden, for example, certain regions use the selective screening approach, i.e. testing women who only meet the high risk criteria, while others use the universal screening approach, i.e. screening all women for GDM [24]. As it is not cost effective to screen every woman most developing countries adopt a selective screening approach and usually only screen women who meet certain GDM risk criteria [24]. In South Africa, different regions follow different practices and there is no set screening protocol. However, most use the selective screening approach, whereby if a woman falls into a high risk category (three risk factors or more) she will have an OGTT performed [24].

Reported risk factors for GDM are maternal age over 25 years, pre-pregnancy body mass index (BMI) above 30 kg/m^2^, a previous macrosomic baby (>4.0 kg), a previous diagnosis of GDM, a first degree relative with either type 1 or type 2 diabetes, and ethnicity (certain ethnic groups have a high prevalence of diabetes e.g. Asian, Indian and African) [25]. From the above list of risk factors the only modifiable risk factor is BMI. However, one important factor that seems to be overlooked in most of the criteria is the physical activity levels and sedentary behaviour of women during pregnancy. This is surprising as physical activity has been shown to assist with weight control during pregnancy thus addressing the only modifiable risk factor [13].

A recent systematic review reported that physical activity interventions during pregnancy have resulted in a reduction in gestational weight gain [26]. Since excessive weight gain has been associated with the development of GDM [27], physical activity may play an important role in reducing risk for GDM. Liu et al. [28] have shown that prenatal exercise is associated with a lower risk for GDM among previously inactive women. Studies have shown that moderate-to-vigorous intensity physical activity (MVPA) before, as well as during pregnancy, has a positive effect on insulin sensitivity, glucose tolerance and an overall reduction in risk for GDM [14, 15].

A longitudinal study conducted by Van Poppel et al. [14] found objectively measured MVPA in obese women resulted in decreased triglycerides and improved insulin response and sensitivity at 32 weeks of pregnancy.

A self-reported measure of PA remains the most cost effective and feasible way of collecting data in large population groups and provides domain-specific activity levels. However, self-reported questionnaires have been shown to over-estimate moderate-to-vigorous intensity physical activity levels and should be complemented with objective measures such as accelerometers [29], which remain the gold standard for measuring PA levels [14, 29]. Therefore, a combination of self-reported and objective measures of PA is more likely to yield the best results.

## Methods/Design

### Aim

The aim of this study is to (1) examine the association between physical activity levels during the second trimester and glucose tolerance as determined by the accelerometer data and OGTT results; (2) examine the association between physical activity levels in the second trimester and insulin sensitivity as determined by metabolic testing conducted; (3) examine the changes in physical activity during the pregnancy in pregnant black South African women living in urban Soweto, South Africa.

### Hypothesis

A higher level of physical activity throughout pregnancy improves glucose tolerance and insulin sensitivity.

### Study objectives

1. To describe the change in physical activity levels and patterns between the second and third trimesters of pregnant women living in Soweto.
2. To determine the association between maternal glucose and insulin concentrations and maternal physical activity levels during the second trimester.

### Study design

This prospective cohort study will selectively include women enrolled into the Soweto First 1000 Days Study (S1000) at the MRC/Wits Departmental Pathways for Health Research Unit (DPHRU) based at the Chris Hani Baragwanath Academic Hospital in Soweto, South Africa.

### Study setting

Soweto is an urban area in Johannesburg, South Africa, with the majority of residents being of black South African descent [30, 31]. All interviews, assessments and procedures will take place at the DPHRU which consists of offices, a laboratory, and examination and interview rooms dedicated solely to research studies. Participants will be seen for their scheduled visits at the site and all measures and samples will be collected there. The on-site laboratory will analyse all the blood samples.

### Sample population

Women enrolled into the S1000 study will be eligible to participate in the sub-study. They will have to meet the following inclusion criteria:

- Women living in the Soweto region
- ≥18 years of age
- < 14 weeks pregnant
- Pregnant with a singleton pregnancy
- Non-diabetic at the time of recruitment

### Sample size

Based on the estimated prevalence (50 %) of active and inactive participants, the sample size has been calculated to reach a confidence level of 95 % with a power of 80 % and α significance level of 0.05. Taking the above calculations into account, 80 participants will need to be recruited into this study. This will be achieved via convenience sampling from the women enrolled into the larger S1000 study. Data for the first 80 women with valid PA measurements and OGTT results will be used.

### Data collection and measures

The outcomes of this study include: time spent in MVPA and sedentary behaviours as measured by self-reported questionnaires and objective accelerometry; the GDM risk factors that are present; positive diagnosis of GDM; gestational weight gain and blood glucose and insulin concentrations from the OGTT (Table 1).

**Table 1:** Outcome measures, timing of data collection and source of data

	Timing	Source
Demographic data	Visit 1: 14–18 weeks gestation	• Demographic data sheet
Assess the presence of established Gestational Diabetes mellitus risk factors	Visit 1: 14–18 weeks gestation	• Demographic data sheet
Assess Physical Activity levels	Visit 1: 14–18 weeks gestation Visit 3: 28–33 weeks gestation	• GPAQ • ActiGraph Accelerometer data
Assess Blood Glucose and insulin Concentrations	Visit 2: 24–28 weeks gestation	• Oral Glucose Tolerance Test (Fasting, 60 min and 120 min Plasma readings)

#### Self-report measure of physical activity and sedentary time

Physical activity and sedentary behaviour will be measured using the validated GPAQ [32]. The questionnaire collects data in four domains of physical activity: 1) work; 2) travel; 3) recreational activities and 4) time spent sitting/reclining. The GPAQ is completed via interview at two time points during pregnancy (Visit 1 and 3). Time spent in each domain (work, travel, recreational and sedentary time) is calculated and using the MET rating for moderate and vigorous activity, an outcome measure of minutes per week (mins/wk) moderate-vigorous physical activity (MVPA) for the various domains and for total PA is then calculated. The Global Physical Activity Questionnaire (GPAQ) was developed by the World Health Organization (WHO) as a means of producing a standardised tool for measuring valid and reliable estimates of physical activity internationally [32]. It is particularly relevant for developing countries as it takes into account the assorted patterns of energy expenditure unique to these populations. Its usage in Africa has been widespread, and it has been validated in nine different developing countries [32].

#### Objective measure of physical activity and sedentary time

Objective physical activity will be measured using an ActiGraph accelerometer (ActiGraph^TM^, Pensacola, FL) which participants will be required to wear for seven consecutive days. The ActiGraph accelerometer is a small (5.1 × 4.1 × 1.5 cm) and light (0.4 kg) instrument, that records integrated acceleration information as an activity count, providing an estimate of the intensity of the body’s movements. Participants will be categorised as active if they are meeting the current recommendations of 150 min per week of moderate intensity physical activity, as stipulated by the American College of Obstetrics and Gynaecologists [33], or inactive if they are not.

Freedson cut points [34] will be used to convert the raw accelerometer data into intensity bands. Active activity is categorised in to four subcategories, light (100–1951counts/min), moderate (1952–5124 counts/min), vigorous (5125–9498 counts/min) and very vigorous (>9498 counts/min). Sedentary time will be measured at <100 count/min. Average time in minutes per day (min/day) will be recorded for sedentary time and physical activity time (light- and MVPA-intensity) [35]. For the data to be considered valid the participants will have to wear the accelerometer at both time points (Table 1), for a minimum of 4 days per week and 10 h of wear-time per day.

#### Blood Glucose Measure (OGTT)

A 75 g 2 h OGTT will be performed on the women when they are between 24 and 28 weeks pregnant (Table 1). Blood glucose (mmol/L) and insulin (pmol/L) samples will be taken at 0 min (fasting), 30, 60 and 120 min post glucose load. These samples will be run in real-time within the DPHRU laboratory. Gestational diabetes mellitus will be diagnosed according to the IADPSG diagnostic criteria, whereby one or more of the following glucose values should be equalled or exceeded: Fasting glucose >5.1 mmol/l; 60 min plasma reading >10.0 mmol/l; 120 min plasma reading >8.5 mmol/l.

Height, weight and body mass index (BMI) will be measured at each time point (Visits1, 2, and 3). A demographic questionnaire will be administrated to all participants at their first visit. This questionnaire includes questions pertaining to the risk factors for GDM, such as maternal age, pre-pregnancy weight, family history of diabetes, previous pregnancy and obstetric information, household inventory, smoking history, marital status and employment status (Table 1).

### Data analysis

The results of participant demographics and baseline outcomes variables will be summarised using descriptive summary measures, expressed as means (standard deviation) or medians (normally inter quartile range) depending on the distribution of the data, and frequency (percent) for categorical variables.

A student *T*-test will be used to compare the PA levels in the second and third trimesters. ANOVA test will be used to compare glucose tolerance and insulin sensitivity between the active and inactive groups. Significance will be assumed at a p value of *p* < 0.05 and confidence intervals of 95 %.

A logistic regression analysis will be conducted to determine the independent relationship between variables and GDM.

Statistical analysis will be performed using STATA (Version12.0 for WINDOWS).

## Discussion

Physical activity during pregnancy has been associated with many favourable maternal and neonatal outcomes. Russo et al.[36], reported in a recent meta-analysis that PA during pregnancy was associated with a 28 % lower risk of GDM . Most of the studies conducted thus far have been in developed countries and few have subjectively and objectively measured the physical activity of the participants at two time points during pregnancy. The majority of studies have found a positive association between physical activity and improved insulin sensitivity [14, 37]. In Africa there are no studies that have measured physical activity, glucose metabolism and the risk for GDM. The current protocol in South Africa for the management of GDM mainly consists of medication and diet, with no physical activity intervention being implemented. The results from this study will enable us to identify the physical activity levels and patterns in this population, which will assist in developing targeted interventions, i.e. the utilisation of physical activity as a treatment modality for GDM, as well as developing much needed, feasible healthcare strategies amongst pregnant black South African women. As the burden on the healthcare system and economy grows daily in South Africa, it is of utmost importance that methods to improve maternal health are considered and implemented.

## Abbreviations

BMI: Body mass index
GDM: Gestational diabetes mellitus
GPAQ: Global physical activity questionnaire
IADPSG: International Association of Diabetes and Pregnancy Study Group
MVPA: Moderate-vigorous-physical-activity
OGTT: Oral glucose tolerance test
PA: Physical activity
SB: Sedentary behaviour
WHO: World Health Organization
